# Blocking circ-CNST suppresses malignant behaviors of osteosarcoma cells and inhibits glycolysis through circ-CNST-miR-578-LDHA/PDK1 ceRNA networks

**DOI:** 10.1186/s13018-021-02427-0

**Published:** 2021-05-07

**Authors:** Rui Hu, Shan Chen, Jianxin Yan

**Affiliations:** 1 Department of Spine Surgery Clinic, The Central Hospital of Enshi Tujia and Miao Autonomous Prefecture, Enshi City, Hubei Province China; 2 Department of Oncology, The Central Hospital of Enshi Tujia and Miao Autonomous Prefecture, Enshi City, Hubei Province China; 3 Department of Joint Surgery, The Central Hospital of Enshi Tujia and Miao Autonomous Prefecture, No. 158 Wuyang Avenue, Enshi City, 445000 Hubei Province China

**Keywords:** Circ-CNST, miR-578, Osteosarcoma, Glycolysis, LDHA, PDK1

## Abstract

**Background:**

CircRNA CNST (circ-CNST) is a newly identified biomarker for prognosis of osteosarcoma (OS). However, its role in OS progression remains to be well documented.

**Methods:**

Expression of circ-CNST, microRNA (miR)-578, lactate dehydrogenase A (LDHA), and pyruvate dehydrogenase kinase 1 (PDK1) was detected by quantitative real-time polymerase chain reaction and Western blotting. The physical interaction was confirmed by dual-luciferase reporter assay. Cell behaviors and glycolysis were measured by 3-(4,5-dimethylthiazolyl-2)-2,5-diphenyltetrazolium bromide assay, colony formation assay, flow cytometry, transwell assays, xenograft experiment, and commercial kits.

**Results:**

Circ-CNST was upregulated in human OS tissues and cells, accompanied with downregulation of miR-578 and upregulation of LDHA and PDK1. There were negative correlations between miR-578 expression and circ-CNST or LDHA/PDK1 in OS tissues. Moreover, high circ-CNST/LDHA/PDK1 or low miR-578 might predict shorter overall survival, advanced TNM stages, and lymph node metastasis. Physically, miR-578 was targeted by circ-CNST, and miR-578 could target LDHA/PDK1. Functionally, blocking circ-CNST and restoring miR-578 enhanced apoptosis rate and suppressed cell proliferation, colony formation, migration, and invasion in 143B and U2OS cells, accompanied with decreased glucose consumption, lactate production, and adenosine triphosphate (ATP)/adenosine diphosphate (ADP) ratio. Furthermore, in vivo growth of U2OS cells was retarded by silencing circ-CNST. Depletion of miR-578 could counteract the suppressive role of circ-CNST deficiency in 143B and U2OS cells, and restoring LDHA or PDK1 partially reversed the role of miR-578 inhibition as well.

**Conclusion:**

Circ-CNST knockdown could antagonize malignant behaviors and glycolysis of OS cells by regulating miR-578-LDHA/PDK1 axes.

**Supplementary Information:**

The online version contains supplementary material available at 10.1186/s13018-021-02427-0.

## Introduction

Osteosarcoma (OS), arising from transformed mesenchymal cells, is the most frequent primary bone tumor ranging from children to adolescents and young adults [1]. Metabolic reprogramming in cancer is manifested by persistent aerobic glycolysis and suppression of mitochondrial function, which is called the Warburg effect, a hallmark of many malignant tumors including OS [2]. Although the generation of adenosine triphosphate (ATP) is less efficient, tumor cells benefit from aerobic glycolysis in growth, metastasis, and drug resistance [3, 4]. Lactate is an energy source and L-lactate shuttles from OS cells to neighboring cells, mediating tumor microenvironment [5, 6]. Although treatment for OS has been improved, the likelihood of survival remains low for OS patients with metastasis and recurrence. Moreover, pulmonary metastasis is observed in 30–35% of OS patients after initial treatment for 2 to 3 years [7]. It is necessary to understand the molecular mechanism of OS progression and glycolysis to identify more effective therapeutic targets.

Very recently, emerging roles of noncoding RNAs have been identified in the pathogenesis, diagnosis, and prognosis of OS [8]. Circular RNAs (circRNAs) are generally novel endogenous noncoding RNAs with covalently closed-loop structure, and deregulation of circRNAs has been demonstrated in OS patients by Gene Expression Omnibus (GEO) database. The regulatory functions of circRNAs result in OS cell progression or restraint [9]. Moreover, circRNAs are attractive new class of potential biomarkers and therapeutic targets for OS [10, 11]. CircRNA CNST (circ-CNST, hsa_circ_0017311) is upregulated in OS patients and represents an independent biomarker for poor prognosis [12]. However, the function and mechanism of circ-CNST in OS progression remains largely unclear, since it has only recently identified in OS.

In this study, we attempted to investigate the role of circ-CNST dysregulation in malignant behaviors of OS cells and glycolysis. Lactate dehydrogenase A (LDHA) and pyruvate dehydrogenase kinase 1 (PDK1) are key players involved in anaerobic glycolysis and tumor progression [13, 14]. Strikingly, LDHA and PDK1 are emerging anti-OS therapeutic targets [15, 16]. Severing as microRNAs (miRNAs) sponge is one of biological functions of circRNAs in various tumors including OS [10, 17]. Furthermore, circRNA-based competing endogenous RNA (ceRNA) networks have been constructed in OS based on circRNA-miRNA pairs and miRNA-messenger RNA (mRNA) pairs [18, 19]. Thus, we aimed to further confirm whether miRNA (miR)-578, a poorly understood miRNA yet, was a target of circ-CNST in regulating LDHA and PDK1.

## Materials and methods

### OS patients and tissue samples

Carcinoma tissues and corresponding adjacent non-carcinoma tissues were obtained from 29 patients with primary OS during the operation. All specimens were diagnosed as OS by clinical, imaging, and histological examinations, and patients received anti-tumor treatment before this study were excluded. Clinicopathologic features of these patients were summarized in Table 1. This study was ratified by the Medical Ethics Committee of the Central Hospital of Enshi Tujia and Miao Autonomous Prefecture, and informed consent was provided form each patient or his guardian.

**Table 1:** Relationship between circ-CNST expression and clinicopathologic features of osteosarcoma patients

	Characteristics, *n* = 29	circ-CNST expression		*P* value^a^
		Low (*n* = 14)	High (*n* = 15)	
Gender				
Female	17	9	8	0.7104
Male	12	5	7	
Age (years)				
≤ 60	15	9	6	0.2723
> 60	14	5	9	
TNM grade				
I + II	12	9	3	0.0253*
III + IV	17	5	12	
Lymph node metastasis				
Positive	18	5	13	0.0078*
Negative	11	9	2	
Tumor size				
≤ 5 cm	11	10	1	0.0016*
> 5 cm	18	4	14	

*TNM*, tumor-node-metastasis

*P < 0.05

^a^Chi-square test

### Cells and cell culture

Human fetal osteoblasts (hFOB1.19; #60357) and human OS cell lines including HOS (#CRL-1543), MG63 (#CRL-1427), 143B (#CRL-8303), and U2OS (#HTB-96) were purchased from BCRC (Taiwan, China) and ATCC (Manassas, VA, USA). The hFOB1.19 cells were cultured in Dulbecco’s modified Eagle’s medium (DMEM)/F12 (R&D systems, Minneapolis, MN, USA) at 34 °C, and OS cells were cultivated in DMEM (R&D systems) at 37 °C. All cells were incubated in medium supplemented with 10% fetal bovine serum with 5% CO_2_.

### Ribonuclease R (RNase R) treatment and quantitative real-time polymerase chain reaction (RT-qPCR)

Cultured cells and tissues were subjected to RNeasy kit (Invitrogen, Carlsbad, CA, USA) following the manufacturer’s instructions to extract total RNA, which was used to synthesize complementary DNA (cDNA) with Reverse Transcription System (Promega, Madison, WI, USA). For RNase R treatment, total RNA (2 μg) from 143B and U2OS cells was incubated with 5 U RNase R (Solarbio, Beijing, China) for 30 min at 37 °C. The expression levels of circ-CNST, linear CNST, miR-578, LDHA, and PDK1 were quantified with GoTaq® qPCR and RT-qPCR systems (Promega) and normalized to β-actin and U6 mRNA levels. The primers were summarized in Table 2. The relative expression was calculated using the 2^−ΔΔCt^ method.

**Table 2:** The primers and siRNAs used in this study

Name	Sequence
circ-CNST (152 nt)	5′-TGTGCAACCACATACAGTTACG-3′ (forward)
	5′-CAGCATATGTGACTGC-3′ (reverse)
linear CNST (165 nt)	5′-TCCCCTTTGCCTTCATCAGA-3′ (forward)
	5′-TGTGGTTGCACAGTTTTCCA-3′ (reverse)
miR-578	5′-GTGCAGGGTGTTAGGA-3′ (forward)
	5′-GAAGAACACGTCTGGT-3′ (reverse)
LDHA (165 nt)	5′-CAGGTGGTTGAGAGGGTCTTT-3′ (forward)
	5′-CTTCAAACGGGCCTCTTCCT-3′ (reverse)
PDK1	5′-GCTTCGATGCAGCAACAACA-3′ (forward)
	5′-AGGCGCTGCTTTAAGCTCTG-3′ (reverse)
β-actin	5′-ACACCTTCTACAATGAGCTG-3′ (forward)
	5′-CTGCTTGCTGATCCACATCT-3′ (reverse)
U6	5′-CTCGCTTCGGCAGCACAT-3′ (forward)
	5′-AACGCTTCACGAATTTGCGT-3′ (reverse)
si(sh)-circ-CNST	5′-GTCTCTTGATGTGTTGCACAA-3′ (sense)
	5′-GTGCAACACATCAAGAGACTT-3′ (anti-sense)
si (sh)-NC	5′-TCTGGTTGACATCATGCTTAATGTG-3′ (sense)
	5′-CATTAAGCATGATGTCAACCAGACA-3′ (anti-sense)
miR-578 mimic	5′-CUUCUUGUGCUCUAGGAUUGU-3′
miR-NC	5′-GGUUCGUACGUACACUGUUCA-3′
anti-miR-578	5′-ACAATCCTAGAGCACAAGAAG-3′
anti-miR-NC	5′-UGAACAGUGUACGUACGAACC-3′

### Cell transfection

Cell transfection of exogenous nucleotides into 143B and U2OS cells was performed with Lipofectamine 3000 reagent (Invitrogen) following the manufacturer’s instructions. Small interference RNA (siRNA) targeting circ-CNST (si-circ-CNST) and its negative control si-NC, short hairpin RNA (shRNA) targeting circ-CNST (sh-circ-CNST) and sh-NC, miR-578 mimic and miR-NC mimic, and miR-578 inhibitor (anti-miR-578) and anti-miR-NC were synthesized by GENEWIZ (Beijing, China). The sequences were presented in Table 2. The shRNAs were inserted into pSilencer 4.1-CMV puro vector (EK-Bioscience, Shanghai, China), and stably shRNA-transfected cells were selected by Puromycin. Overexpression vectors of circ-CNST, LDHA, and PDK1 were constructed depending on pCD5-ciR (Geneseed, Guangzhou, China) and pcDNA3.1 (+) (EK-Bioscience). The transiently transfected cells were collected after transfection for 24 h.

### 3-(4,5-dimethylthiazolyl-2)-2,5-diphenyltetrazolium bromide (MTT) assay and colony formation assay

For MTT assay, transfected cells 143B and U2OS cells were re-seeded in 96-well plate at a density of 2 × 10^4^ cells per well, and each group was set with 6 parallel wells. The cells were cultured with complete medium for another 24 h, 48 h, and 72 h, and subjected to MTT Cell Proliferation and Cytotoxicity Assay Kit (Beyotime, Shanghai, China) according to the directions for use. The colorimetric analysis was measured at 570 nm on Multiskan Ascent 354 microplate reader (Abcam, Cambridge, MA, USA).

For colony formation assay, 143B and U2OS cells were re-seeded in 6-well plate after transfection for 24 h at a density of 200 cells per well. Three parallel wells were set up in each transfection group. The cells were cultured in complete medium for 14 days. The colonized spots were stained with 0.25% crystal violet solution (Beyotime). The colonies were observed under a microscope, and colony number was counted.

### Flow cytometry (FCM)

Annexin V-fluorescein isothiocyanate (FITC) apoptosis detection kit (Beyotime) and FCM were performed to measure apoptosis rate of 143B and U2OS cells with different transfection. A total of 5 × 10^4^ cells were harvested and re-suspended in Annexin V-FITC binding buffer. Then, 5 μL of Annexin-FITC and 10 μL of propidium iodide (PI) were added in sequence and kept away from light for 15 min. Stained cells were examined on FACS Calibur flow cytometer(BD Biosciences, San Jose, CA, USA) and analyzed on FACS Diva (BD Biosciences).

### Transwell assays

Cell migration and invasion were tested by transwell insert (6.4-mm diameter and 8-μm pore size) purchased from BD Biosciences. For migration assay, the lower chamber was added with complete medium, and the upper insert was added with 5 × 10^4^ cells in serum-free medium. Cells in transwell system were cultured for another 48 h, and then subjected to crystal violet (0.25%) staining. Stained cells were observed under a microscope (× 100), and migratory cell number was counted. For invasion assay, the insert was coated with Matrigel membrane (BD Biosciences), and 5 × 10^5^ transfected cells were used.

### Determination of glucose, lactate, and ATP levels

Glucose Uptake Assay Kit (Colorimetric), L-Lactate assay Kit (Colorimetric), and adenosine diphosphate (ADP)/ATP Ratio Assay Kit (Bioluminescent) were provided from Abcam (Cambridge, UK). All experiments were conducted in accordance with the manuals, respectively.

### Xenograft tumor models

All animal experiments were reviewed and approved by the Institutional Animal Care and Use Committee of the Central Hospital of Enshi Tujia and Miao Autonomous Prefecture. A sum of 16 female ALB/c nude mice (4-week-old) were purchased and maintained under pathogen-free conditions. Serum-free cell suspensions of stably transfected U2OS cells were injected subcutaneously into the posterior flanks of nude mice at a density of 5 × 10^6^ cells per mice, and the mice were randomly divided into two groups (*n* = 8): sh-NC and sh-circ-CNST. Caliper was used to measure tumor dimensions every 3 days after 7-day inoculation, and volume was calculated according to 0.5 × length × width^2^ formula. At the termination of the experiment, mice were sacrificed and tumors were excised. Xenograft tumors were weighted with electronic scales and photographed with a camera. Tumor tissues were stored in liquid nitrogen for further RNA isolation. All animal experiments were performed following the Guide for the Care and Use of Laboratory Animals (GB/T 35892-2018; Standardization Administration of the People Republic of China).

### Dual-luciferase reporter assay

Circinteractome (https://circinteractome/circular_rna_query=hsa_circ_0017311&mirna) and Targetscan (http://www.targetscan/vert_71/=hsa-miR-578) databases were utilized to predict miR-578-binding sites in circ-CNST, LDHA, and PDK1. To test the abovementioned target relationships, dual-luciferase reporter assays were carried out. The wild-type (WT) sequences (with a specific binding site for miR-578) of circ-CNST, LDHA 3′UTR, and PDK1 3′UTR were respectively cloned into luciferase reporter vector pMIR-REPORT (Promega, containing Firefly), as well as the mutated (MUT) sequences (with mutations of miR-578-binding sites). 143B and U2OS cells were co-transfected with WT/MUT vectors, pRL-SV40 (internal control vector, containing Renilla), and miR-578 mimic or miR-NC mimic. After transfection for 24 h, cell lysate was collected and luciferase activities of Firefly and Renilla were measured by the Dual-Luciferase Reporter Assay Kit (Promega) on GloMax Discover Microplate Reader (Promega).

### Western blotting

Total protein in cultured cells was extracted by RIPA Lysis Buffer (Beyotime), and protein concentration was measured using BCA Protein Assay Kit (Beyotime). Proteins were denatured in loading buffer by boiling at 100 °C for 10 min, and denatured protein samples were subjected to Western blotting assay [20]. The antibodies used were presented in Table 3. ECL™ Western blotting system (Merck, Darmstadt, Germany) was used to detect protein signals and densitometric quantification was performed using ImageJ software (NIH, Bethesda, MD, USA).

**Table 3:** The antibodies used in this study

Name	Cat. no.	Dilution ratio	Source
LDHA	AF0216	1:1000	Beyotime
PDK1	AF7707	1:1000	Beyotime
β-actin	AA128	1:1000	Beyotime
HRP-labelled anti-Rabbit IgG	A0208	1:1000	Beyotime
HRP-labelled anti-Mouse IgG	A0216	1:1000	Beyotime

### Statistical analysis

Statistical data were presented as means (± standard deviation) from at least three independent experiments, and data analysis was further carried out using Student’s *t*-test and/or one-way analysis of variance followed with Tukey’s post hoc test. The statistical analysis was determined by GraphPad Prism 5.0 software (GraphPad, San Diego, CA, USA). Kaplan-Meier survival analysis determined overall survival of 29 OS patients with high/low expression of circ-CNST, miR-578, LDHA, and PDK1. A comparison with *P* < 0.05 was deemed as statistically significant: asterisk represented *P* < 0.05, double asterisks represented *P* < 0.01, triple asterisks represented *P* < 0.001, and quadruple asterisk represented *P* < 0.0001.

## Results

### Circ-CNST was an upregulated circRNA in OS patients and cells

**Fig. 1:**
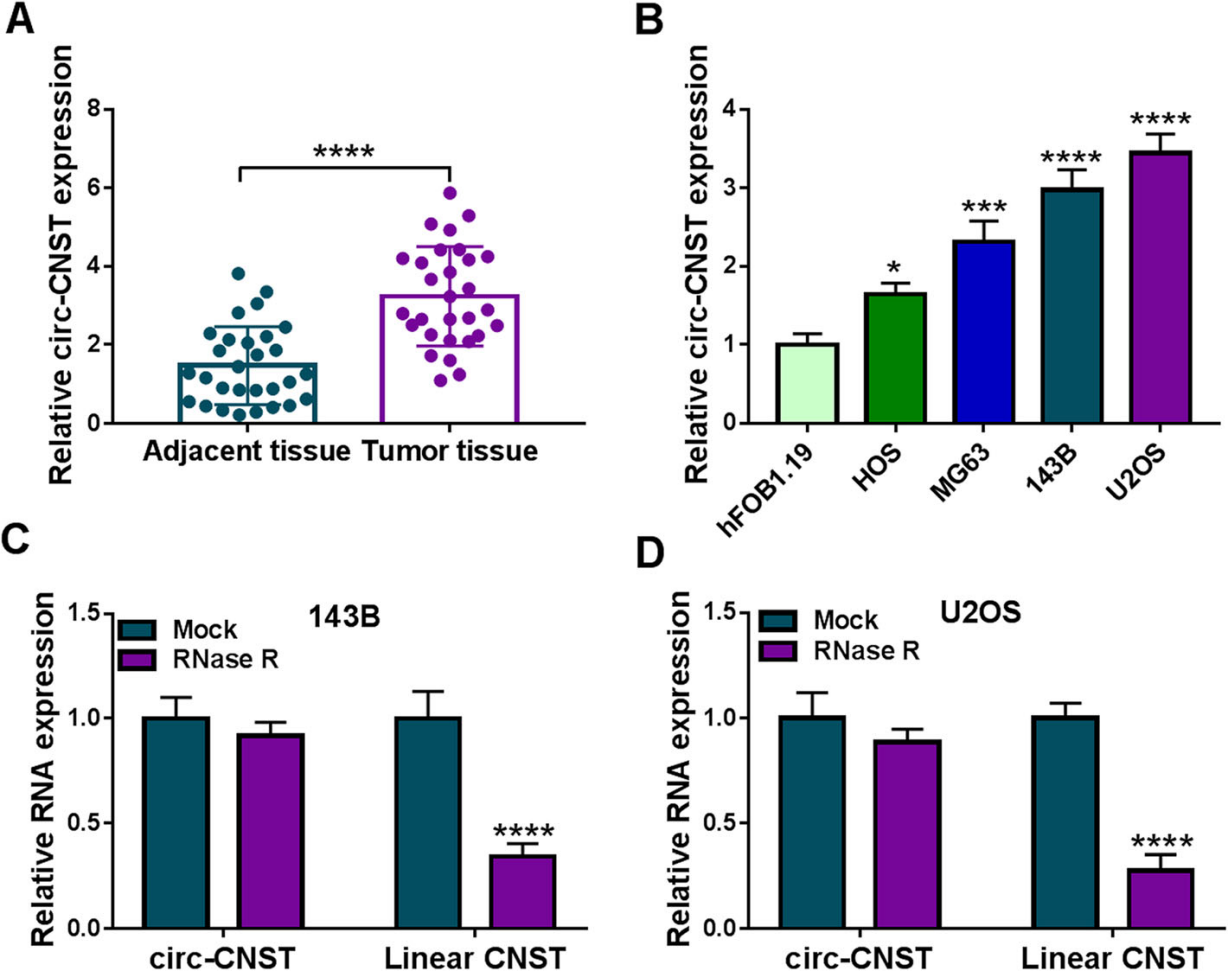
Expression of circ-CNST in OS patients and cells. **a**, **b** RT-qPCR measured relative circ-CNST expression in tumor tissues from OS patients (*n* = 29) comparing to adjacent normal tissues, and in human OS cell lines (HOS, MG63, 143B, and U2OS) comparing to hFOB1.19 cell line. **c**, **d** RT-qPCR measured relative RNA expression of circ-CNST and linear CNST in 143B and U2OS cells treated with RNase R paralleled with mock-treated cells. *P < 0.05, ***P < 0.001, and ****P < 0.0001

Even though expression of circ-CNST in OS had once been identified, it was further validated in this present study. A set of OS patients were enrolled, and RT-qPCR data indicated that relative circ-CNST expression was increased in tumor tissues than adjacent normal tissues (Fig. 1a). Besides, high circ-CNST was found in OS tumors from patients with shorter overall survival, advanced TNM stages, and lymph node metastasis (Figure S1A-S1C). Moreover, Chi-square test confirmed that circ-CNST level was significantly correlated with clinicopathologic features of OS patients, including TNM stage, lymph node metastasis, and tumor size (Table 1). In vitro, its level was higher in several OS cell lines, and 143B and U2OS cells exhibited the highest level of circ-CNST (Fig. 1b). The structure stability was examined by RNase R digestion. Linear CNST expression was significantly attenuated, but circ-CNST expression was little affected in RNase R-treated 143B and U2OS cells (Fig. 1c, d). These results indicated that circ-CNST was a stably upregulated circRNA in OS and might predict heavy tumor burden and poor prognosis.

### Depleting circ-CNST antagonized malignant behaviors and glycolysis of OS cells

**Fig. 2:**
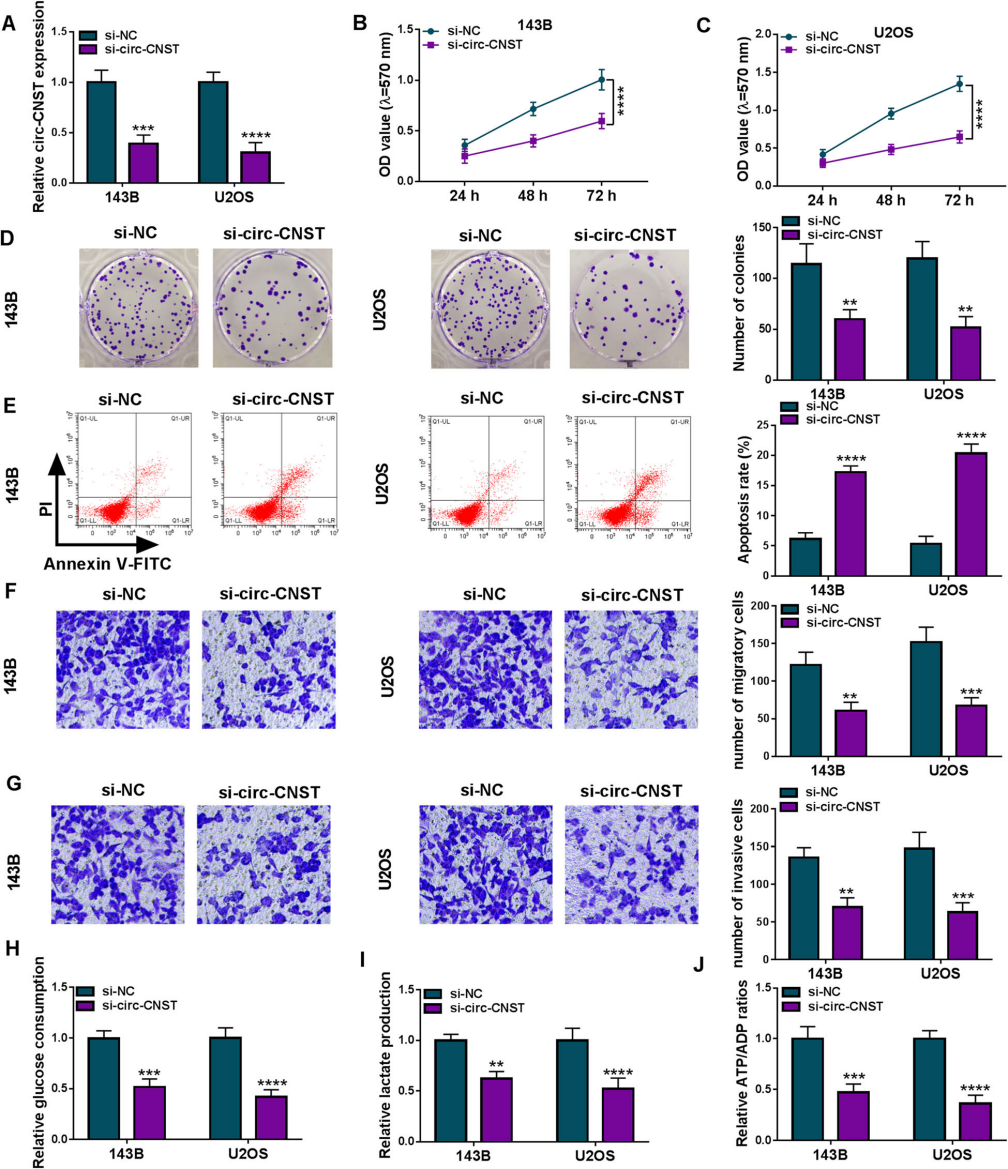
Role of circ-CNST deficiency in cell behaviors and glycolysis of OS cells in vitro. 143B and U2OS cells were transfected with si-circ-CNST for 24 h comparing to si-NC-transfected cells. **a** RT-qPCR detected relative circ-CNST expression. **b**, **c** MTT assay measured optical density (OD) value at 570 nm at indicated time-points. **d** Colony formation assay evaluated number of colonies. **e** FCM determined apoptosis rate (%). **f**, **g** Transwell assay examined numbers of migratory cells and invasive cells. **h**–**j** Corresponding kits severally tested glucose consumption, lactate production, and ATP/ADP ratio. **P < 0.01, ***P < 0.001, and ****P < 0.0001

Loss-of-function experiments were subsequently carried out to testify the role of circ-CNST in OS development. Special siRNA was utilized to knock down the abnormally high expression of circ-CNST in OS cells, and circ-CNST level was dramatically decreased in si-circ-CNST-transfected 143B and U2OS cells (Fig. 2a). Cell proliferation analyzed by MTT assay was inhibited by circ-CNST knockdown in transfected 143B and U2OS cells, as depicted by lower OD values (Fig. 2b, c); similarly, colony formation inhibition was observed in 143B and U2OS cells transfected with si-circ-CNST, as indicated by the declined number of colonies (Fig. 2d). On the contrary, apoptosis rate of 143B and U2OS cells was elevated with si-circ-CNST transfection than si-NC transfection, suggesting a promoting role of circ-CNST knockdown in OS cell apoptosis*.* Transwell assays showed less migratory cells and invasive cells after transfection of si-circ-CNST versus si-NC in both 143B and U2OS cells (Fig. 2f, g). Furthermore, commercial kits revealed an overall reduction of glucose consumption, lactate production, and ATP/ADP ratio in circ-CNST-depleted 143B and U2OS cells (Fig. 2h–j). Collectively, these results demonstrated a tumor-suppressive role of circ-CNST knockdown in human OS cells in vitro by inhibiting malignant behaviors and glycolysis.

**Fig. 3:**
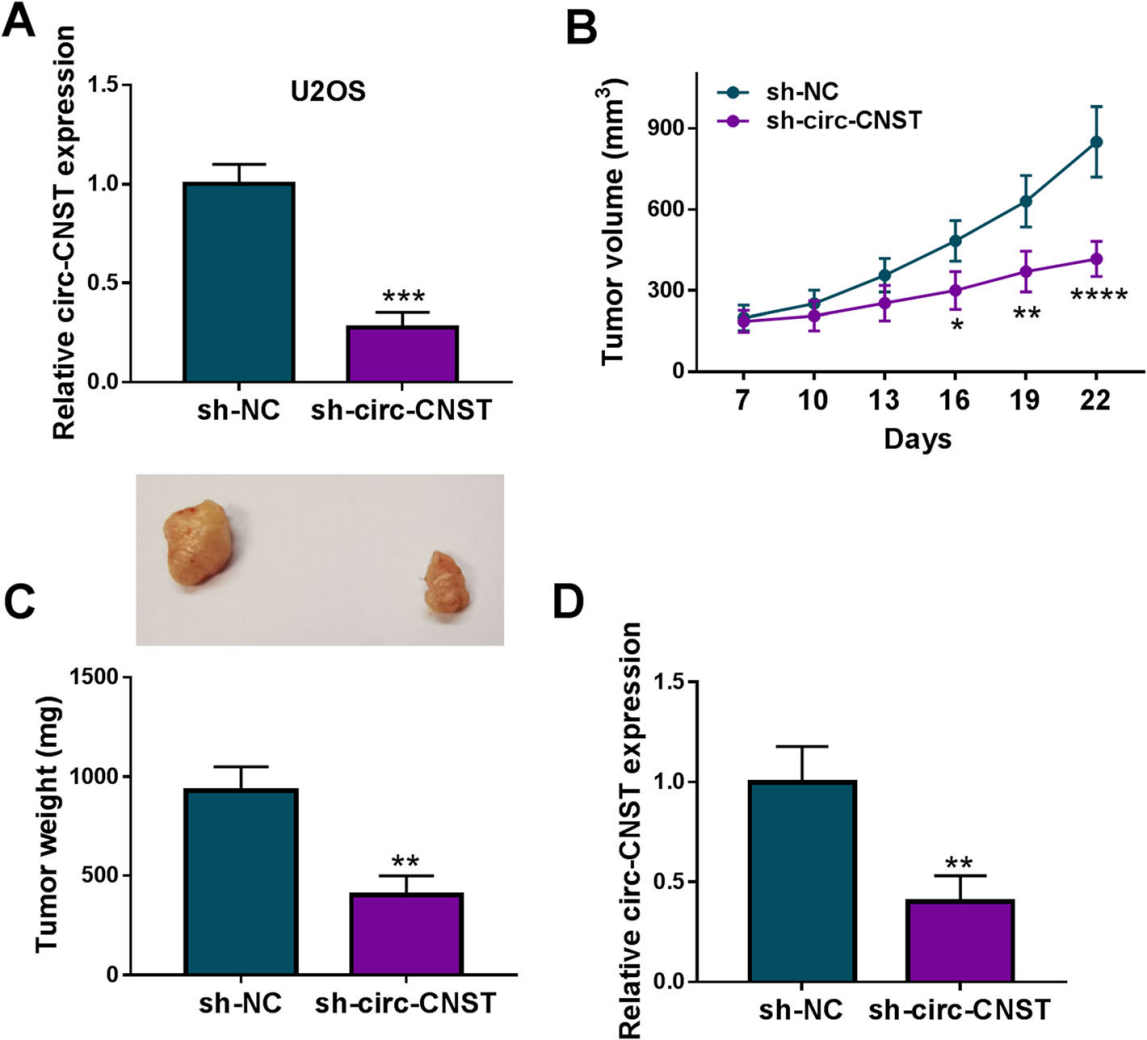
Role of circ-CNST deficiency in cell growth of OS cells in vivo. **a** RT-qPCR detected relative circ-CNST expression in U2OS cells stably transfected with sh-circ-CNST comparing to sh-NC-transfected cells. **b**, **c** Transfected U2OS cells were inoculated in nude mice, and 8 mice were set in each group. **b** Tumor volume was calculated at indicated time-points. **c** Tumor weight was measured on the last day. **d** RT-qPCR detected relative circ-CNST expression in tissues from xenograft tumors. *P < 0.05, **P < 0.01, ***P < 0.001, and ****P < 0.0001

Thereafter, sh-circ-CNST was used to stably block circ-CNST expression in U2OS cells (Fig. 3a), as well as 143B cells (Data were not showed). Comparing to 143B cells, U2OS cells showed higher circ-CNST level and higher transfection efficiency of sh-circ-CNST and thus stably transfected U2OS cells were injected into nude mice to induce xenograft tumors. As a result, the volume and weight of xenograft tumors were restrained by blocking circ-CNST in advance (Fig. 3b, c), and lower circ-CNST level was detected in tumor tissues in sh-circ-CNST group (Fig. 3d). This result demonstrated a cell growth inhibition of circ-CNST deficiency in OS in vivo.

### Circ-CNST negatively modulated miR-578 in human OS cells via target binding

**Fig. 4:**
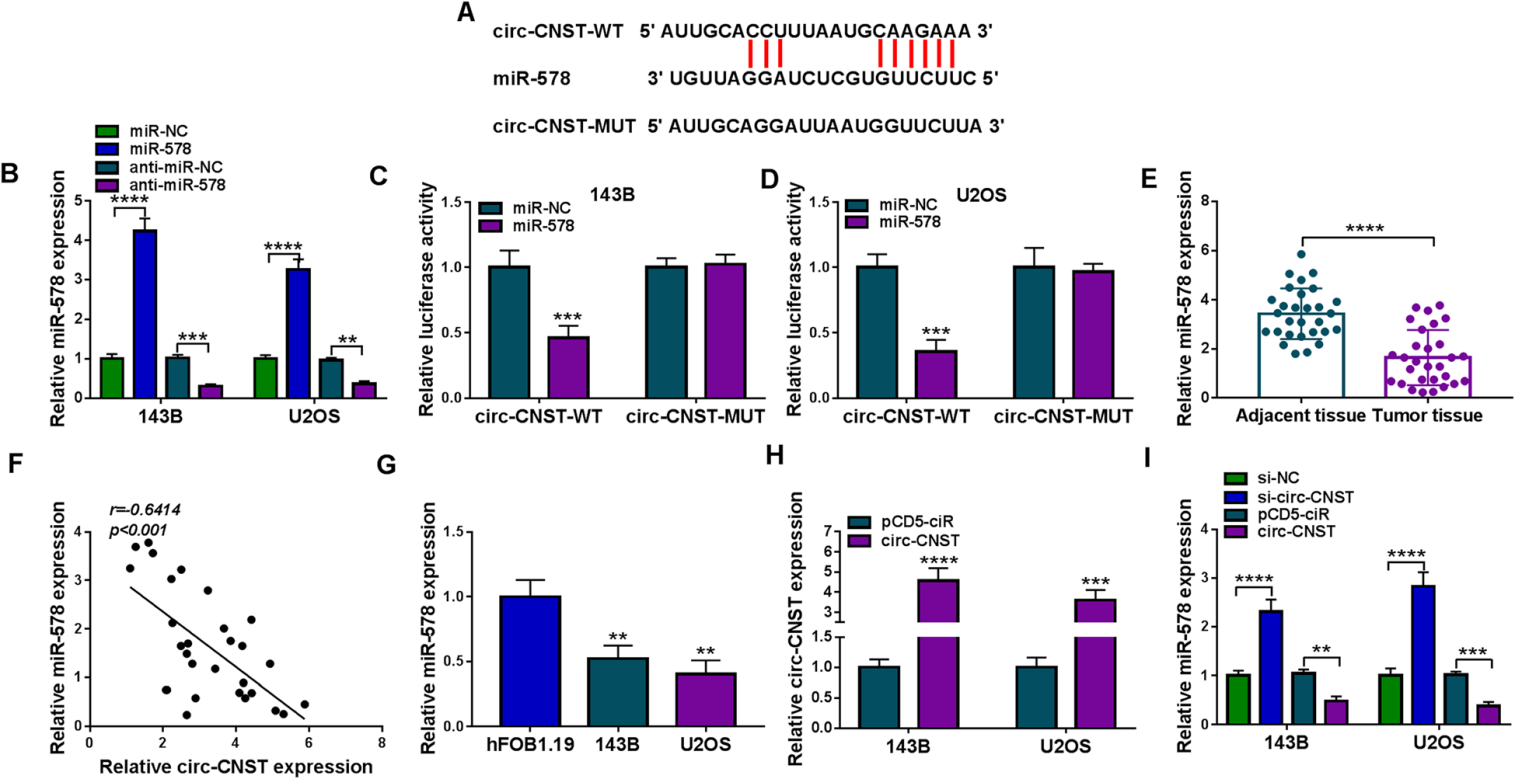
The relationship of circ-CNST with miR-578. **a** Sequences were aligned between miR-578 and circ-CNST-WT or circ-CNST-MUT. **b** RT-qPCR detected relative miR-578 expression in 143B and U2OS cells transfected with miR-578 mimic (miR-578), miR-NC mimic (miR-NC), anti-miR-578, or anti-miR-NC. **c**, **d** Dual-luciferase reporter assay identified relative luciferase activity of 143B and U2OS cells co-transfected with WT/MUT vector and miR-578 or miR-NC. **e** RT-qPCR detected relative miR-578 expression in tumor tissues from OS patients (*n* = 29) comparing to adjacent normal tissues. **f** Pearson correlation coefficient analysis validated the linear correlation between circ-CNST and miR-578 expression in human OS tumors. **g** RT-qPCR detected relative miR-578 expression in 143B and U2OS cells comparing to hFOB1.19 cells. **h**, **i** RT-qPCR detected relative circ-CNST and miR-578 expression in 143B and U2OS cells transfected with circ-CNST vector, empty pCD5-ciR vector, si-circ-CNST, or si-NC. **P < 0.01, ***P < 0.001, and ****P < 0.0001

The circinteractome database was selected to annotate miRNA targeting sites in circ-CNST, and miR-578 was predicted to be complementary to circ-CNST (Fig. 4a). MiR-578 mimic transfection led to overexpression of miR-578 in both 143B and U2OS cells (Fig. 4b), which attenuated the luciferase activity of circ-CNST-WT vector and failed to affect luciferase activity of circ-CNST-MUT vector (Fig. 4c, d). This data validated the binding relationship between circ-CNST and miR-578. Expression of miR-578 was detected by RT-qPCR, and its level was lower in OS patients’ tumor tissues and cells (Fig. 4e, g). Moreover, Pearson correlation coefficient analysis determined that miR-578 expression was negatively correlated with circ-CNST in OS tumors (Fig. 4f). Besides, low miR-578 was also found in OS tumors from patients with shorter overall survival, advanced TNM stages and lymph node metastasis (Figure S2A-S2C). In 143B and U2OS cells, transfection of pCD5-ciR-circ-CNST (circ-CNST) vector induced ectopic expression of circ-CNST (Fig. 4h), and allied with that was the downregulation of miR-578 (Fig. 4i); contrarily, silencing circ-CNST resulted in miR-578 upregulation (Fig. 4i). These data demonstrated that miR-578 was downregulated in OS patients and cells and was negatively regulated by circ-CNST via target binding.

### Downregulating miR-578 counteracted the suppressive role of circ-CNST deficiency in OS cells in vitro

**Fig. 5:**
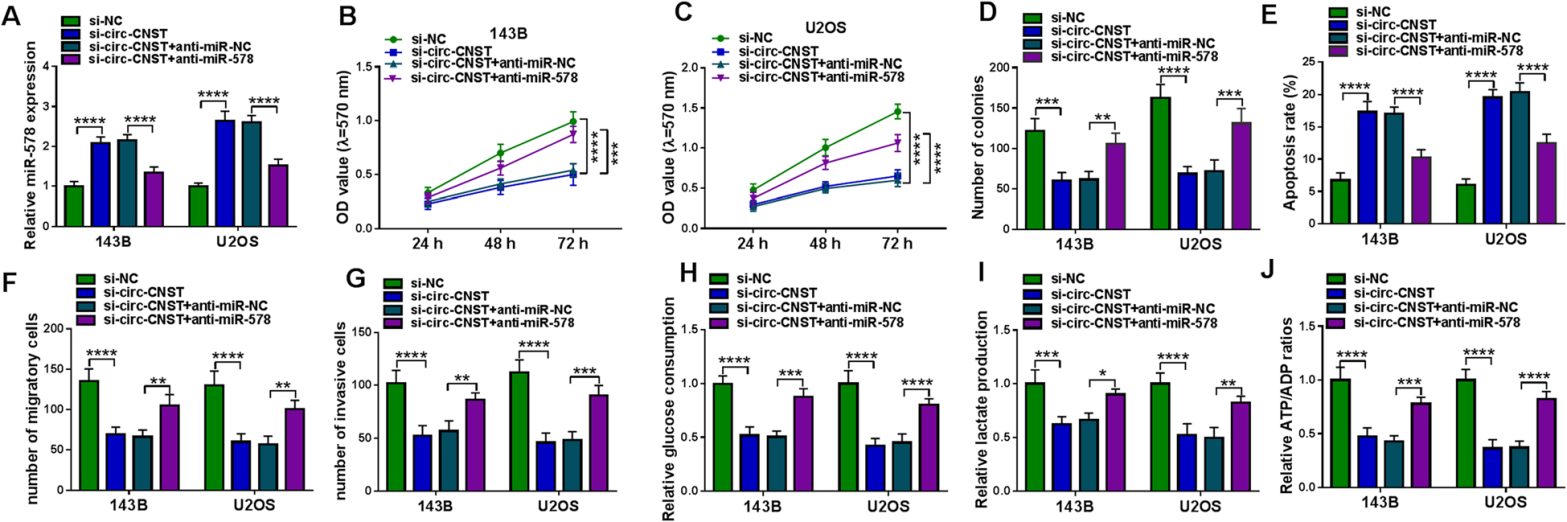
Effect of miR-578 downregulation on role of circ-CNST deficiency in human OS cells in vitro*.* 143B and U2OS cells were transfected with si-circ-CNST comparing to si-NC-transfected cells, and co-transfected with si-circ-CNST and anti-miR-578 paralleled with cells co-transfected with si-circ-CNST and anti-miR-NC. **a** RT-qPCR detected relative miR-578 expression. **b**, **c** MTT assay measured OD value at indicated time-points. **d** Colony formation assay evaluated number of colonies. **e** FCM determined apoptosis rate (%). **f**, **g** Transwell assay examined numbers of migratory cells and invasive cells. **h**–**j** Corresponding kits severally tested glucose consumption, lactate production, and ATP/ADP ratio. *P < 0.05, **P < 0.01, ***P < 0.001, and ****P < 0.0001

Anti-miR-578 transfection could lead to depletion of miR-578 in 143B and U2OS cells without and with circ-CNST knockdown (Figs. 4b and 5a). Furthermore, in circ-CNST-silenced 143B and U2OS cells, the inhibited cell proliferation and colony formation were improved with anti-miR-578 transfection, as indicated by higher OD values and colony number (Fig. 5b, d). Additional anti-miR-578 transfection diminished apoptosis rate of 143B and U2OS cells expressing si-circ-CNST (Fig. 5e). Silencing circ-CNST depressed the numbers of transwell migratory cells and invasive cells in 143B and U2OS cells, and this depression was attenuated by simultaneously depleting miR-578 (Fig. 5f, g). Glycolysis of 143B and U2OS cells was inhibited when circ-CNST was knocked down, which was rescued in the presence of anti-miR-578, as evidenced by the increase of glucose consumption, lactate production and ATP/ADP ratio (Fig. 5h–j). These outcomes depicted the counteractive effect of miR-578 downregulation on the suppressive role of circ-CNST knockdown in malignant behaviors and glycolysis of human OS cells in vitro, suggesting a circ-CNST-miR-578 pair in OS.

### Overexpressing miR-578 suppressed malignant behaviors and glycolysis of OS cells in vitro through inhibiting its target genes, LDHA and PDK1

**Fig. 6:**
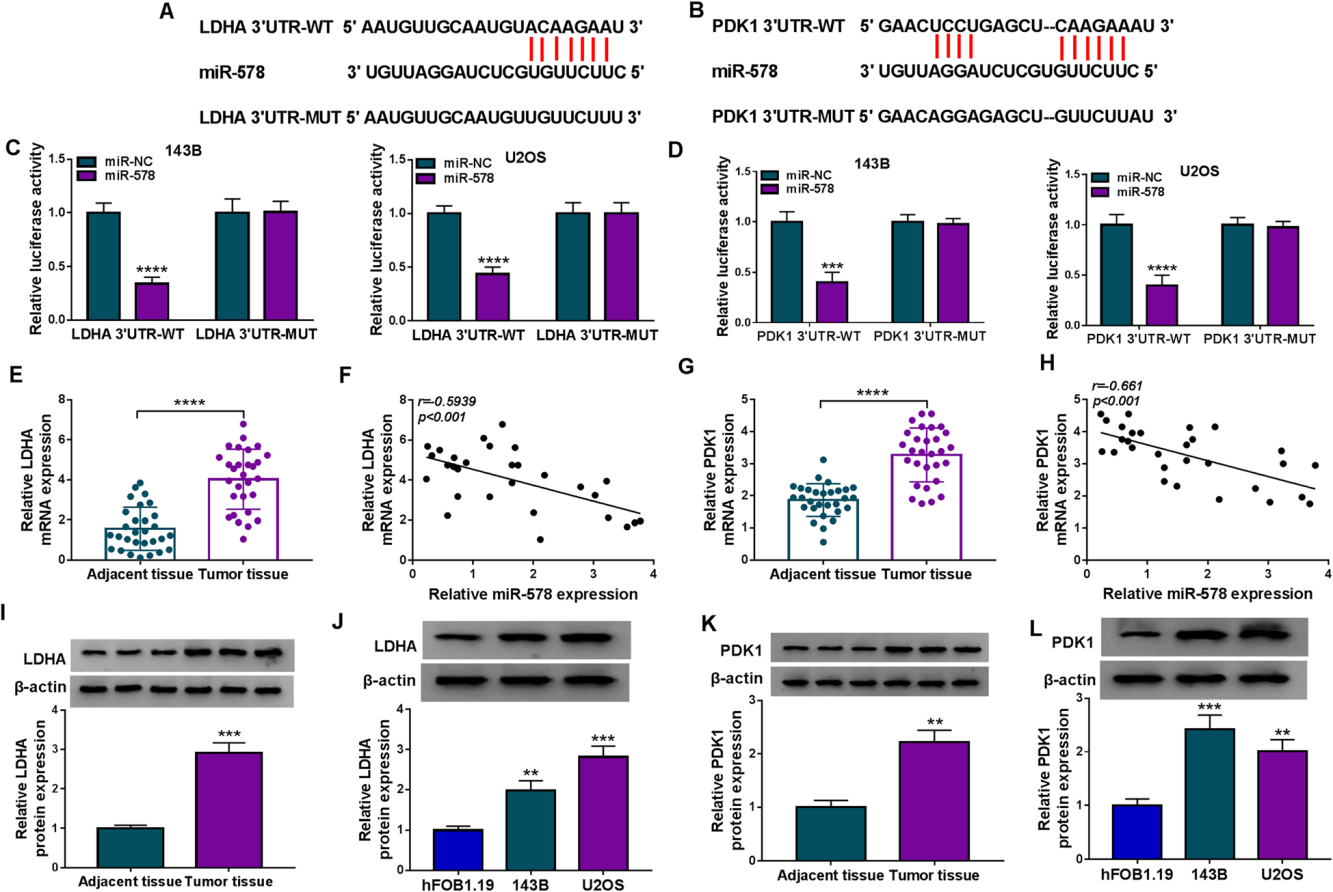
The downstream targets of miR-578 in human OS cells. **a**, **b** Alignment sequences between miR-578 and LDHA 3′UTR-WT/MUT or PDK1 3′UTR-WT/MUT. **c**, **d** Dual-luciferase reporter assay identified relative luciferase activity of 143B and U2OS cells co-transfected with WT/MUT vector and miR-578 or miR-NC. **e**, **g** RT-qPCR detected relative LDHA and PDK1 mRNA expression in tumor tissues from OS patients (*n* = 29) comparing to adjacent normal tissues. **f**, **h** Pearson correlation coefficient analysis validated the linear correlation between miR-578 expression and mRNA expression of LDHA or PDK1 in human OS tumors. **i**–**l** Western blotting measured relative protein expression of LDHA and PDK1 in OS patients’ tumor tissues and adjacent tissues, and cell lines including 143B, U2OS cells, and hFOB1.19 cells. **P < 0.01, ***P < 0.001, and ****P < 0.0001

**Fig. 7:**
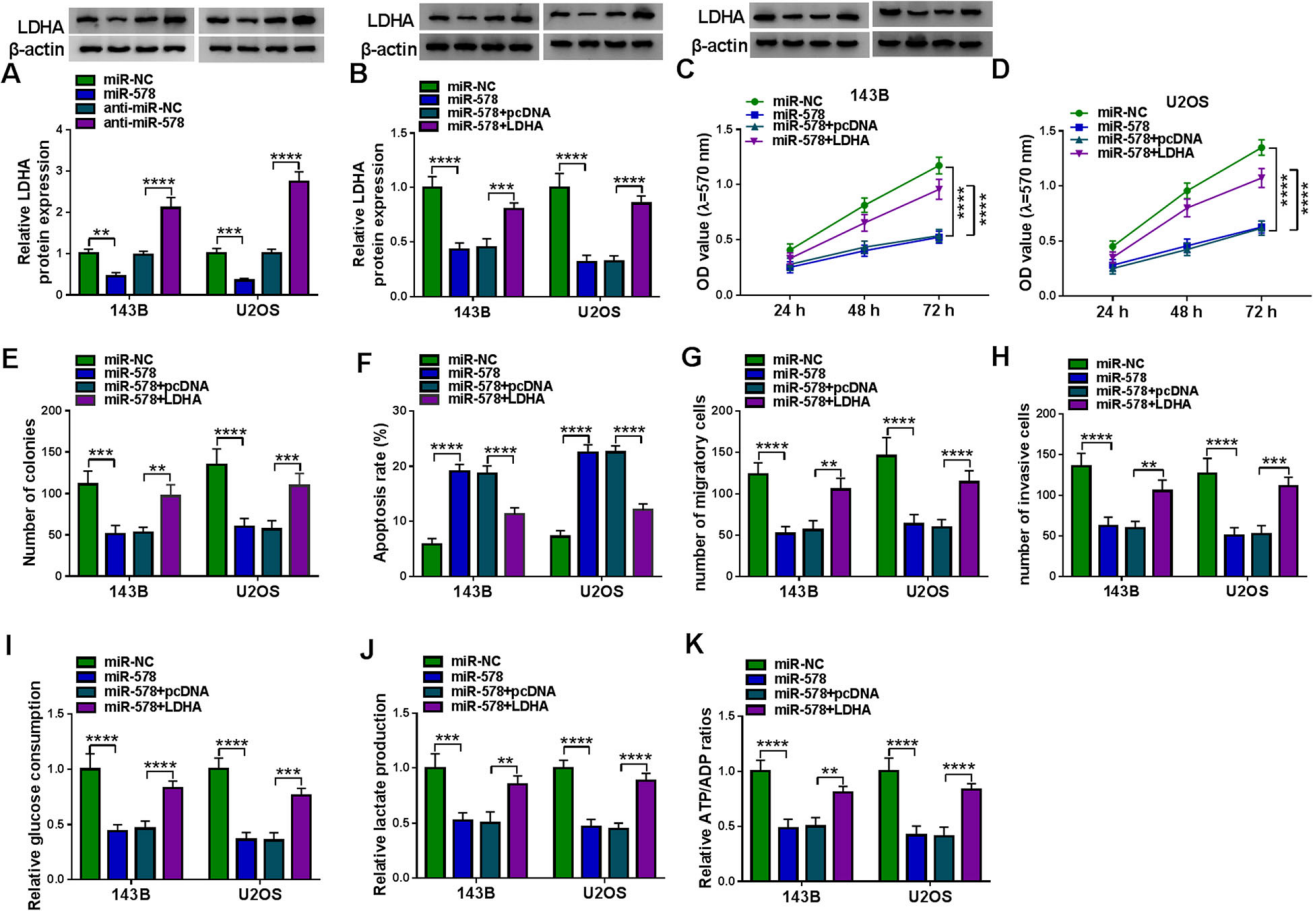
Mutual effect between miR-578 and LDHA in cell behaviors and glycolysis of OS cells in vitro. **a** Western blotting detected relative LDHA protein expression in 143B and U2OS cells transfected with miR-578, miR-NC, anti-miR-578, or anti-miR-NC. **b**–**k** 143B and U2OS cells were transfected with miR-578 comparing to miR-NC-transfected cells and co-transfected with miR-578 and pcDNA-LDHA (LDHA) or pcDNA-PDK1 (PDK1) vector paralleled with cells co-transfected with miR-578 and empty pcDNA vector (pcDNA). **b** Western blotting detected relative LDHA protein expression. **c**, **d** MTT assay measured OD value at indicated time-points. **e** Colony formation assay evaluated number of colonies. **f** FCM determined apoptosis rate (%). **g**, **h** Transwell assay examined numbers of migratory cells and invasive cells. **i**–**k** Corresponding kits severally tested glucose consumption, lactate production, and ATP/ADP ratio. *P < 0.05, **P < 0.01, ***P < 0.001, and ****P < 0.0001

**Fig. 8:**
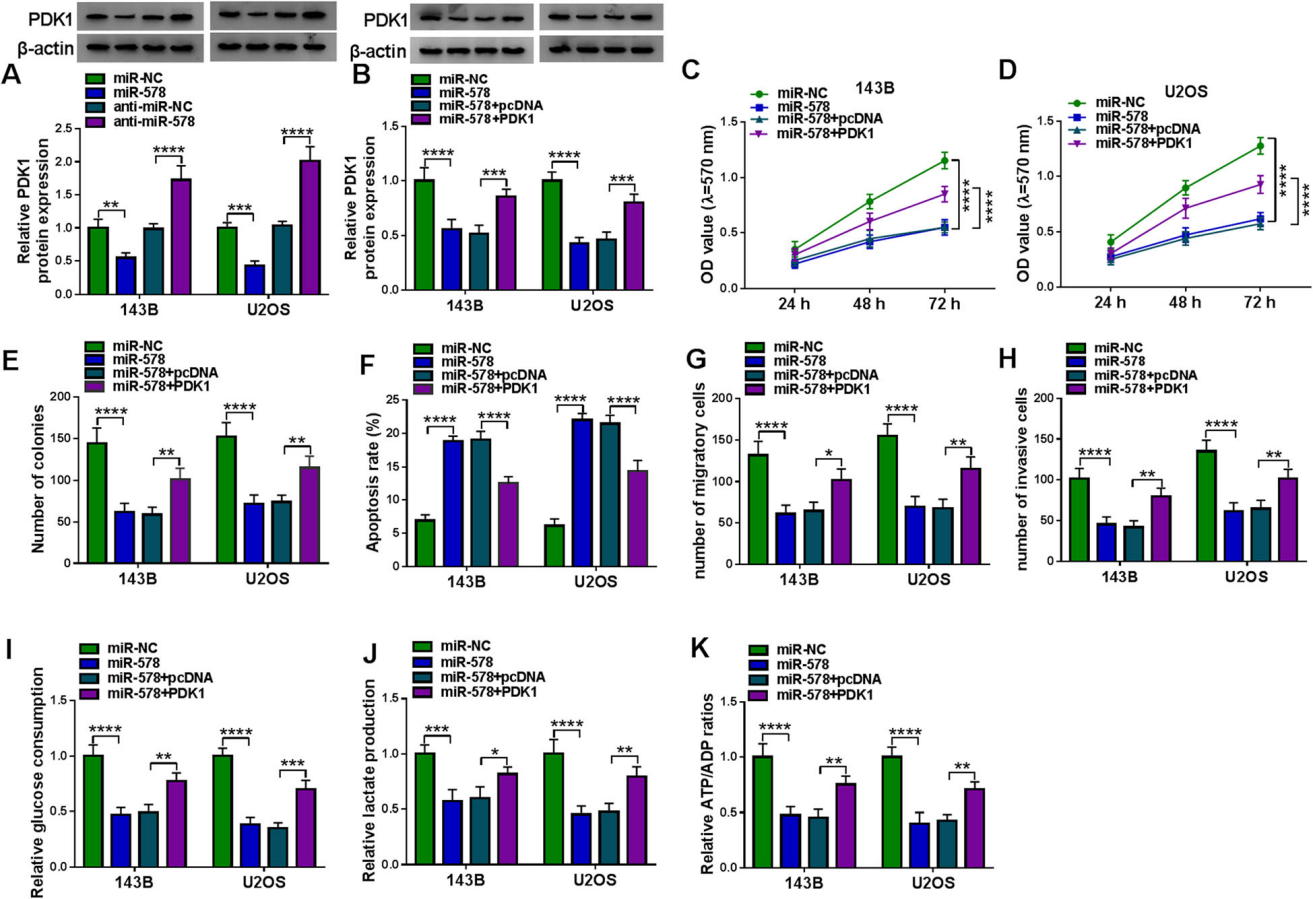
Mutual effect between miR-578 and PDK1 in cell behaviors and glycolysis of OS cells in vitro. **a** Western blotting detected relative PDK1 protein expression in 143B and U2OS cells transfected with miR-578, miR-NC, anti-miR-578, or anti-miR-NC. **b**–**k** 143B and U2OS cells were transfected with miR-578 comparing to miR-NC-transfected cells, and co-transfected with miR-578 and pcDNA-PDK1 (PDK1) vector paralleled with cells co-transfected with miR-578 and pcDNA. **b** Western blotting detected relative PDK1 protein expression. **c**, **d** MTT assay measured OD value at indicated time-points. **e** Colony formation assay evaluated number of colonies. **f** FCM determined apoptosis rate (%). **g**, **h** Transwell assay examined numbers of migratory cells and invasive cells. **i**–**k** Corresponding kits severally tested glucose consumption, lactate production, and ATP/ADP ratio. *P < 0.05, **P < 0.01, ***P < 0.001, and ****P < 0.0001

Targetscan database predicted the potential target genes of miR-578, and glycolysis-related genes were screened. As a results, 3′UTR of LDHA (ENST00000227157.4) and PDK1 (ENST00000282077.3) both showed complementary sequences to miR-578 (Fig. 6a, b). Dual-luciferase reporter assay also identified the reduction of luciferase activity in 143B and U2OS cells co-transfected with LDHA/PDK1 3′UTR-WT vector and miR-578 mimic (Fig. 6c, d). LDHA and PDK1 mRNAs were highly expressed in OS patients’ tumors with negatively linear correlation with miR-578 (Fig. 6e–h). Besides, high LDHA/PDK1 mRNA was found in OS tumors from patients with shorter overall survival, advanced TNM stages, and lymph node metastasis (Figure S3A-S3F). In regarding to protein expression, LDHA and PDK1 levels were higher in three human OS tissues and two OS cell lines (Fig. 6i–l). Expression of LDHA and PDK1 in 143B and U2OS cells was inhibited under miR-578 overexpression condition and was promoted under miR-578 deficiency condition (Figs. 7a and 8a). Notably, LDHA and PDK1 levels in miR-578-overexpressed 143B and U2OS cells could be severally restored by transfecting the recombinant pcDNA plasmids (Figs. 7b and 8b). The overexpression of miR-578 inhibited OD values and colony numbers in 143B and U2OS cells (Figs. 7c–e and 8c–e). On the contrary, apoptosis rate of 143B and U2OS cells was elevated with miR-578 mimic transfection than miR-NC mimic transfection (Fig. 7f and 8f). Transwell migration and invasion were consistently inhibited in both 143B and U2OS cells after transfection of miR-578 mimic, as evidenced by the declined numbers of migratory cells and invasive cells (Figs. 7g, h and 8g, h). Glucose consumption, lactate production, and ATP/ADP ratio were overall decreased in miR-578-upregulated 143B and U2OS cells (Figs. 7i–k and 8i–k). These results demonstrated a tumor-suppressive role of miR-578 in cell proliferation, colony formation, apoptosis, migration, invasion, and glycolysis of human OS cells in vitro, whereas this tumor suppression was partially reversed by ectopic expression of LDHA or PDK1 (Figs. 7c–k and 8c–k). These outcomes suggested a miR-578-LDHA/PDK1 pair in OS.

### Circ-CNST could regulate LDHA and PDK1 expression via sponging miR-578

**Fig. 9:**
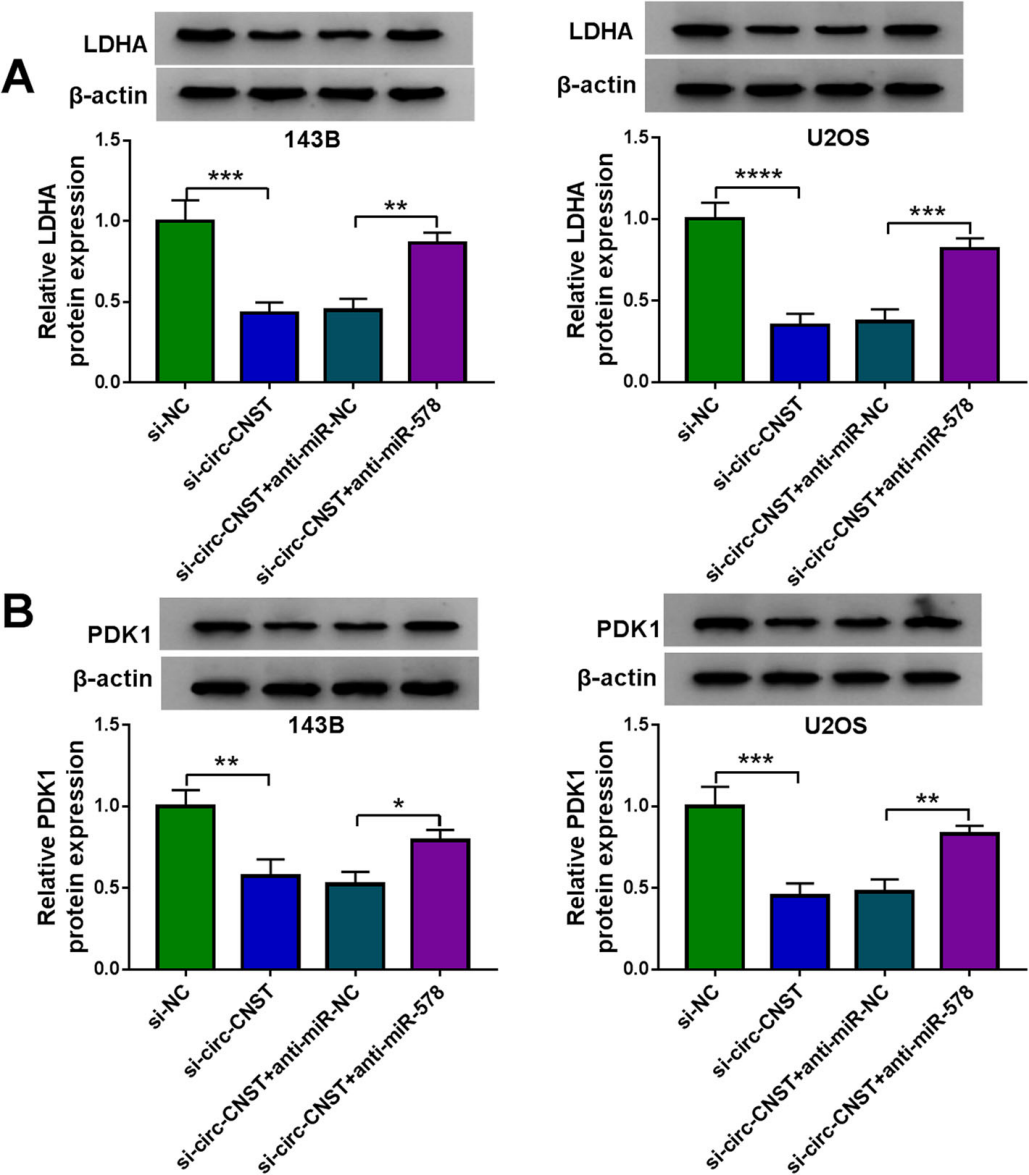
The regulatory effect among circ-CNST, miR-578, and LDHA or PDK1. **a**–**d** Western blotting testified relative LDHA and PDK1 protein expression in 143B and U2OS cells transfected with si-NC or si-circ-CNST, and co-transfected with si-circ-CNST and anti-miR-NC or anti-miR-578. *P < 0.05, **P < 0.01, ***P < 0.001, and ****P < 0.0001

In mechanism, LDHA could be modulated and downregulated by circ-CNST knockdown, and this downregulation was canceled by simultaneously silencing miR-578 (Fig. 9a). Similar to LDHA expression, PDK1 expression could be positively regulated by circ-CNST via miR-578 (Fig. 9b). These outcomes proposed a circ-CNST-miR-578-LDHA/PDK1 ceRNA regulatory network.

## Discussion

Expression profiles of circRNAs had been obtained in human OS tissues [21], as well as OS cell lines [22, 23]. Through analyzing the GSE96964 microarray, several circRNAs had been found to be highly expressed in OS patients’ tumors, such as circ-0000285, circ-XPR1, and circ-0000073 [24–26], as well as circ-CNST [12]. Wang et al. [12] proposed that circ-CNST promoted proliferation and colony formation of OS cells (SW-1353, MG-63, HOS, and U-2OS), and was correlated with tumor size (≥ 5 cm) and shorter survival. Here, except for the inhibitory effect on proliferation and colony formation, we also noticed that circ-CNST knockdown exerted promoting effect on apoptosis and suppressive effect on migration, invasion, and glycolysis in OS cells in vitro. Moreover, growth of OS cells in vivo was retarded by silencing circ-CNST. High circ-CNST expression might predict not only larger tumor size and shorter survival, but also lymph node metastasis in OS patients. Therewith, this study enhanced the understanding of circ-CNST expression and role in OS. By the way, circ-CNST was a cytoplasmic circRNA in OS cells which was resistant to RNase R digestion. Another report also claimed the stability of circ-CNST with actinomycin D treatment [12].

In this study, circ-CNST acted as miR-578 sponge to sequester and inhibit miR-578 expression and activity. Expression of miR-578 was downregulated in OS patients and cells, and abnormal upregulation of miR-578 could inhibit the proliferation and migration of OS cells, which was consistent with previous data [21]. Besides, colony formation, invasion, and glycolysis were observed in this study to be depressed by elevating miR-578 in OS cells, accompanied with apoptosis promotion. Above cell functions of miR-578 in OS cells were similar to that in breast cancer cells [27]. By the way, miR-578 was probably firstly documented to be potential player in BRCA-mutated breast cancer and was associated with VEGF and HIF1 signaling pathways [28]. VEGF and HIF1A were targeted and regulated by miR-578 in regulation of tumor progression [21, 27]. LDHA and PDK1 were key enzymes involved in HIF1 signaling pathway and glycolysis. HIF1A could bind to LDHA, and blocking HIF1A attenuated LDHA expression even under hypoxia [29]. Ku80 was a potential molecular target for melanoma treatment and regulated anti-tumor role of melatonin by targeting and activating PDK1 in HIF1A-dependent manner [30]. Here, we identified LDHA and PDK1 as novel target genes of miR-578 in OS. Both of them drove glycolysis in OS cells to facilitate tumor maintenance partially through miRNAs regulation [31–34]. Our results suggested miR-578 as a novel glycolysis-related miRNA in OS.

Overexpression of LDHA and PDK were observed in several tumors and were frequently associated with chemotherapy-related drug resistance, invasion and metastasis [14, 35, 36]. LDHA was an important part of the final step of the glycolytic pathway, and PDK1 was a gatekeeper enzyme involved in altered glucose metabolism towards glycolysis. Here, we found that expression of LDHA and PDK1 was upregulated in OS tissues and cells, which supported the existing studies [31, 34]. Inhibiting endogenous LDHA and LDHA inhibitors could significantly suppress glycolysis, cell proliferation, and invasion but induce apoptosis of OS cells [15, 32, 37]. Li et al. stated that restoration of PDK1 enhanced proliferative and invasive abilities in OS cells [34]. In addition, colony formation, migration, and glycolysis could also been augmented by overexpressing PDK1, as indicated in this study.

## Conclusion

In conclusion, we demonstrated that silencing circ-CNST could suppress malignant behaviors and glycolysis of OS cells by regulating LDHA and PDK1 via sponging miR-578, suggesting a circ-CNST-miR-578-LDHA/PDK1 ceRNA network in OS. This study enhanced the understanding of circ-CNST role in OS progression and indicated circ-CNST as a potential therapeutic target for OS.

## Supplementary Information

**Additional file 1 **: **Figure S1.** The association between circ-CNST expression and clinical features. (A) Kaplan-Meier survival analysis determined overall survival of 29 OS patients with low expression of circ-CNST (*n*=14) and high expression of circ-CNST (*n*=15). (B, C) RT-qPCR compared relative circ-CNST expression in I-II stage tumors (*n*=12) and III-IV stage tumors (*n*=17), tumors from OS patients with lymph node metastasis (*n*=18) and patients without that (*n*=11). ****P<0.0001.

**Additional file 2 **:**Figure S2.** The association between miR-578 expression and clinical features. (A) Kaplan-Meier survival analysis determined overall survival of 29 OS patients with low expression of miR-578 (*n*=15) and high expression of miR-578 (*n*=14). (B, C) RT-qPCR compared relative miR-578 expression in I-II stage tumors (*n*=12) and III-IV stage tumors (*n*=17), tumors from OS patients with lymph node metastasis (*n*=18) and patients without that (*n*=11). ****P<0.0001.

**Additional file 3 **:**Figure S3.** The association between LDHA/PDK1 expression and clinical features. (A, D) Kaplan-Meier survival analysis determined overall survival of 29 OS patients with low expression of LDHA (*n*=14), high expression of LDHA (*n*=15), low expression of PDK1 (*n*=14), high expression of PDK1 (*n*=15). RT-qPCR compared relative LDHA mRNA and PDK1 mRNA expression in (B, E) I-II stage tumors (*n*=12) and III-IV stage tumors (*n*=17), and (C, F) tumors from OS patients with lymph node metastasis (*n*=18) and patients without that (*n*=11). ****P<0.0001.

## Data Availability

All data generated or analyzed during this study are included in this article.

## Abbreviations

OS: Osteosarcoma
LDHA: Lactate dehydrogenase A
PDK1: Pyruvate dehydrogenase kinase 1
ATP/ADP: Adenosine triphosphate/adenosine diphosphate
GEO: Gene Expression Omnibus
circ-CNST: Circular RNA CNST
ceRNA: Competing endogenous RNA
RT-qPCR: Quantitative real-time polymerase chain reaction
RNase R: Ribonuclease R
cDNA: Complementary DNA
siRNA: Small interference RNA
si-circ-CNST: siRNA targeting circ-CNST
anti-miR-578: miR-578 inhibitor
MTT: 3-(4,5-Dimethylthiazolyl-2)-2,5-diphenyltetrazolium bromide
FCM: Flow cytometry
FITC/PI: Fluorescein isothiocyanate/propidium iodide
