# Supplementary figures and images for: Blocking circ-CNST suppresses malignant behaviors of osteosarcoma cells and inhibits glycolysis through circ-CNST-miR-578-LDHA/PDK1 ceRNA networks
Source: J Orthop Surg Res. 2021 May 7;16:300. doi: 10.1186/s13018-021-02427-0 (PMC8103765; doi:10.1186/s13018-021-02427-0)

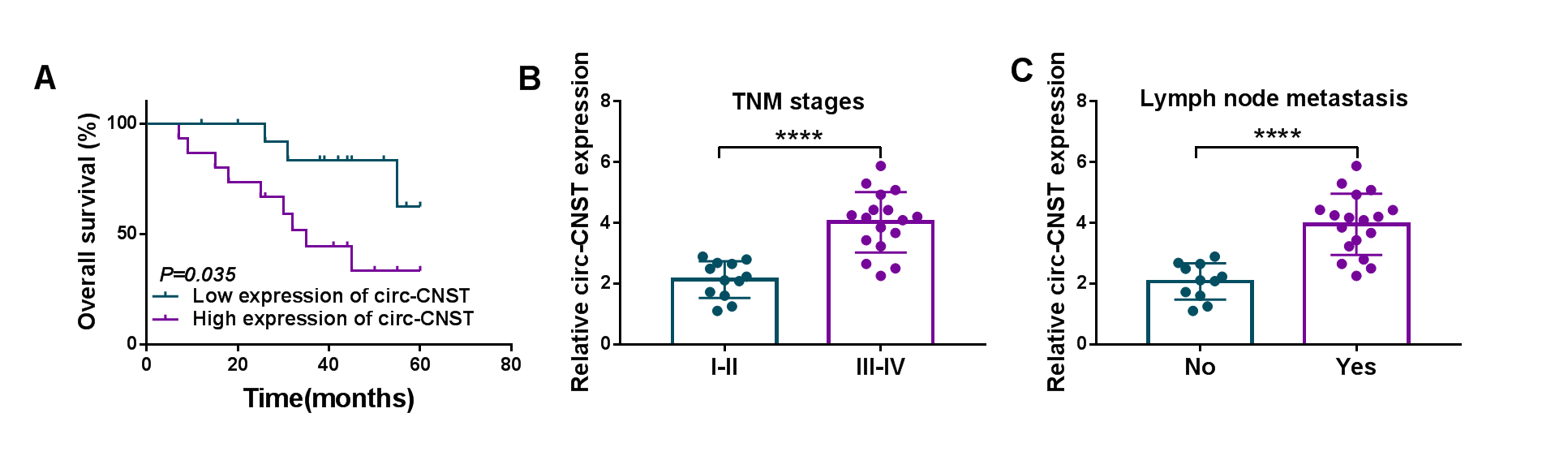

Supplement: Supplementary file 1 — Additional file 1 : Figure S1. The association between circ-CNST expression and clinical features. (A) Kaplan-Meier survival analysis determined overall survival of 29 OS patients with low expression of circ-CNST (n=14) and high expression of circ-CNST (n=15). (B, C) RT-qPCR compared relative circ-CNST expression in I-II stage tumors (n=12) and III-IV stage tumors (n=17), tumors from OS patients with lymph node metastasis (n=18) and patients without that (n=11). ****P<0.0001. [file 13018_2021_2427_MOESM1_ESM.tif]

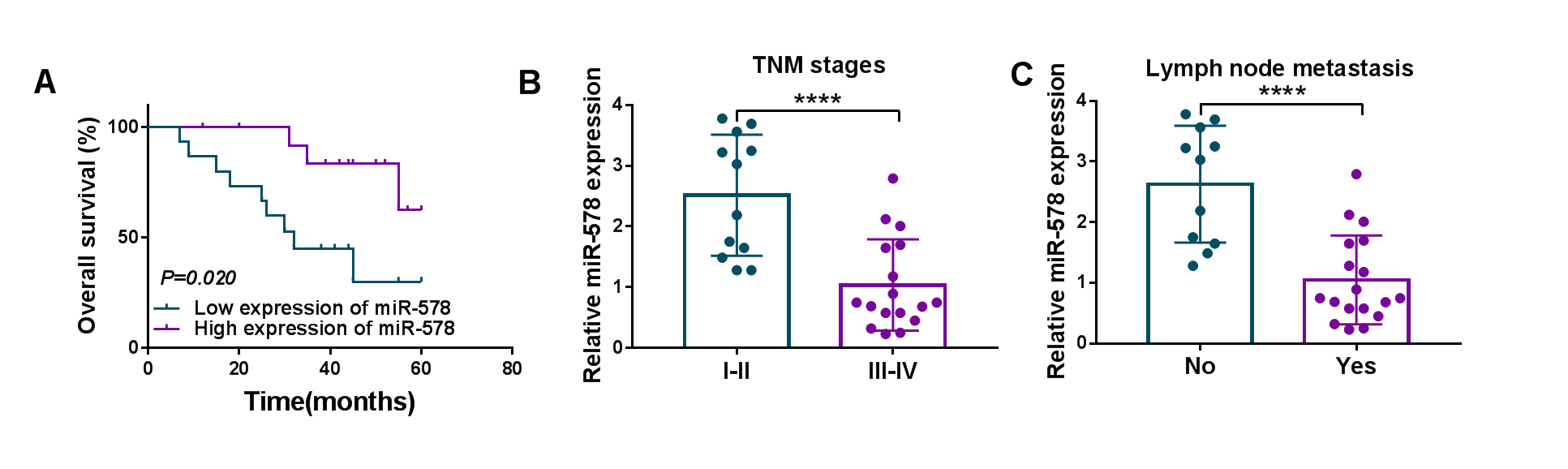

Supplement: Supplementary file 2 — Additional file 2 :Figure S2. The association between miR-578 expression and clinical features. (A) Kaplan-Meier survival analysis determined overall survival of 29 OS patients with low expression of miR-578 (n=15) and high expression of miR-578 (n=14). (B, C) RT-qPCR compared relative miR-578 expression in I-II stage tumors (n=12) and III-IV stage tumors (n=17), tumors from OS patients with lymph node metastasis (n=18) and patients without that (n=11). ****P<0.0001. [file 13018_2021_2427_MOESM2_ESM.tif]

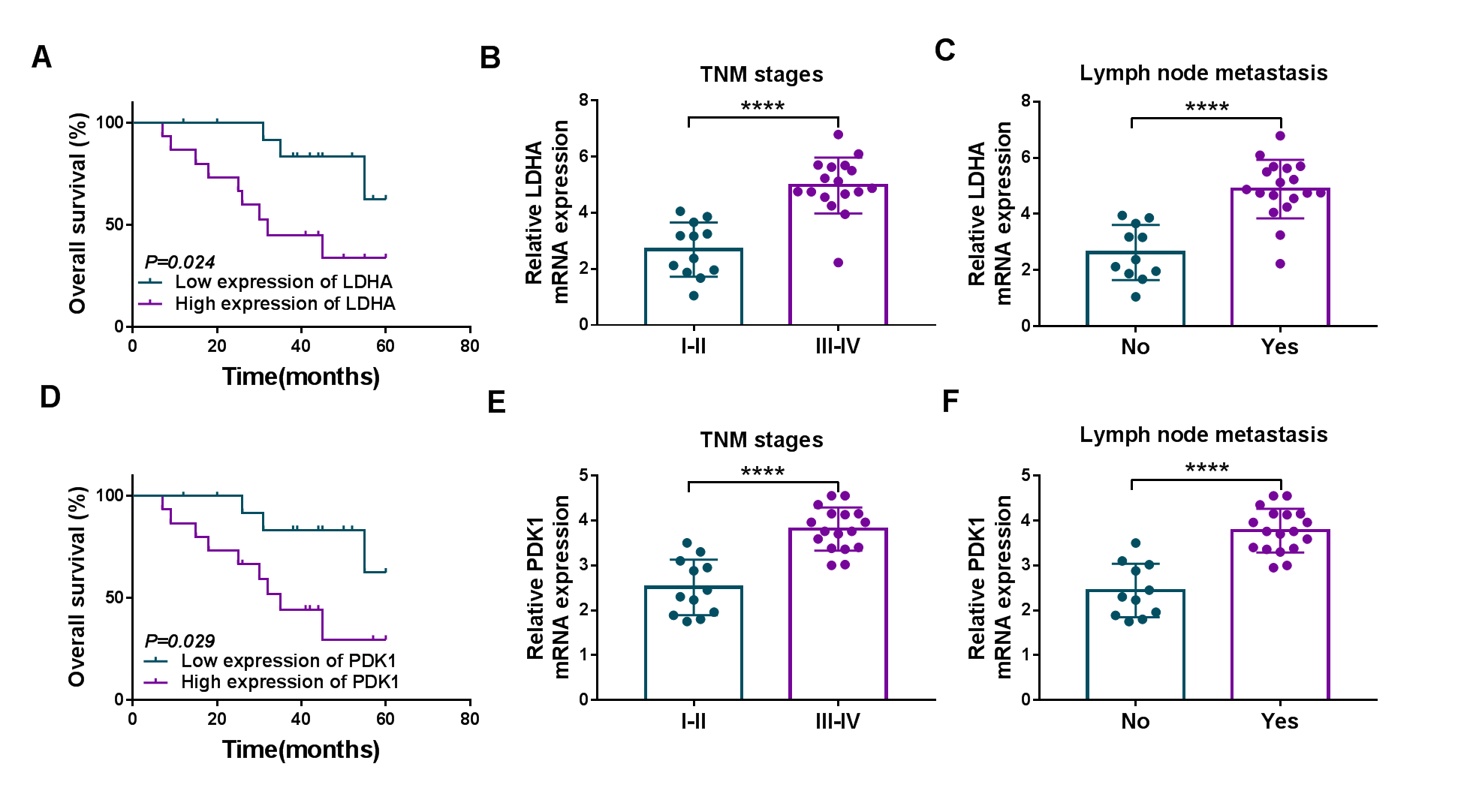

Supplement: Supplementary file 3 — Additional file 3 :Figure S3. The association between LDHA/PDK1 expression and clinical features. (A, D) Kaplan-Meier survival analysis determined overall survival of 29 OS patients with low expression of LDHA (n=14), high expression of LDHA (n=15), low expression of PDK1 (n=14), high expression of PDK1 (n=15). RT-qPCR compared relative LDHA mRNA and PDK1 mRNA expression in (B, E) I-II stage tumors (n=12) and III-IV stage tumors (n=17), and (C, F) tumors from OS patients with lymph node metastasis (n=18) and patients without that (n=11). ****P<0.0001. [file 13018_2021_2427_MOESM3_ESM.tif]
